# Prevalence of *plasmodium falciparum *in active conflict areas of eastern Burma: a summary of cross-sectional data

**DOI:** 10.1186/1752-1505-1-9

**Published:** 2007-09-05

**Authors:** Adam K Richards, Linda Smith, Luke C Mullany, Catherine I Lee, Emily Whichard, Kristin Banek, Mahn Mahn, Eh Kalu Shwe Oo, Thomas J Lee

**Affiliations:** 1 Department of Internal Medicine, Montefiore Medical Center, Albert Einstein College of Medicine, 305 East 161^st ^Street, Bronx, USA 10451; 2 Global Health Access Program, Mae Sot, Thailand; 3 Planet Care/Global Health Access Program, 801 Cedar Street Suite 200, Berkeley, CA, USA 94710; 4 Center for Public Health and Human Rights, Johns Hopkins Bloomberg School of Public Health, 615 N. Wolfe Street, Baltimore, USA 21205; 5 The MENTOR Initiative-Liberia, Monrovia, Liberia; 6 15806 East Saratoga Place Aurora, CO 80015 USA; 7 Backpack Health Worker Team, 659, Moo 1 – Thasailuad, Mae Sot, Tak, Thailand, 63110; 8 Karen Department of Health and Welfare, No. 663 Moo 1 – Thasailuad, Asia High Way, Mae Sot, Tak, Thailand 63110; 9 Department of Medicine, University of California at Los Angeles, 924 Westwood Blvd. Suite 300, Los Angeles, CA, USA 90024

## Abstract

**Background:**

Burma records the highest number of malaria deaths in southeast Asia and may represent a reservoir of infection for its neighbors, but the burden of disease and magnitude of transmission among border populations of Burma remains unknown.

**Methods:**

*Plasmodium falciparum *(*Pf*) parasitemia was detected using a HRP-II antigen based rapid test (Paracheck-Pf^®^). *Pf *prevalence was estimated from screenings conducted in 49 villages participating in a malaria control program, and four retrospective mortality cluster surveys encompassing a sampling frame of more than 220,000. Crude odds ratios were calculated to evaluate *Pf *prevalence by age, sex, and dry vs. rainy season.

**Results:**

9,796 rapid tests were performed among 28,410 villagers in malaria program areas through four years (2003: 8.4%, 95% CI: 8.3 – 8.6; 2004: 7.1%, 95% CI: 6.9 – 7.3; 2005:10.5%, 95% CI: 9.3 – 11.8 and 2006: 9.3%, 95% CI: 8.2 – 10.6). Children under 5 (OR = 1.99; 95% CI: 1.93 – 2.06) and those 5 to 14 years (OR = 2.24, 95% CI: 2.18 – 2.29) were more likely to be positive than adults. Prevalence was slightly higher among females (OR = 1.04, 95% CI: 1.02 – 1.06) and in the rainy season (OR = 1.48, 95% CI: 1.16 – 1.88). Among 5,538 rapid tests conducted in four cluster surveys, 10.2% were positive (range 6.3%, 95% CI: 3.9 – 8.8; to 12.4%, 95% CI: 9.4 – 15.4).

**Conclusion:**

Prevalence of *plasmodium falciparum *in conflict areas of eastern Burma is higher than rates reported among populations in neighboring Thailand, particularly among children. This population serves as a large reservoir of infection that contributes to a high disease burden within Burma and likely constitutes a source of infection for neighboring regions.

## Background

There exists an acute imperative to improve infectious disease surveillance in the border regions of Burma. The combination of multi-drug resistant *plasmodium falciparum *(*Pf*), [1,2] ubiquitous fake antimalarials, [3,4] and under funding of malaria control within a health system ranked 190^th ^out of 191 countries by the WHO in 2000, results in more malaria deaths (1,707) in Burma than any other country in southeast Asia (52.6% of WHO South East Asia Region) [5]. Official statistics are likely to grossly underestimate the number of malaria cases and deaths, especially in remote areas where ongoing civil conflict likely increases malaria risk [6,7]. The most recent WHO country report for Burma provides a striking example of underreporting of malaria morbidity in Karen (Kayin) State. In the same year (2003) that WHO recorded 2,016 malaria cases for the entire state, the Karen Department of Health and Welfare (KDHW) and mobile medics of the Backpack Health Worker Team (BPHWT) treated 27,000 cases in a population of fewer than 300,000 internally displaced persons in Karen State. Furthermore, the Mae Tao Clinic, located across the border from Karen State in Thailand treated over 5,000 confirmed cases of malaria from Burma [8].

Poor malaria control in Burma likely contributes to malaria transmission in neighbouring countries [9-12]. The Thai province of Tak, adjacent to Karen state, has the highest numbers of cases of malaria in the country, and recorded more than twice as many cases (9,339) among Burmese migrants as among Thai locals (4,420) in 2001 [10]. Malaria prevalence in Burmese migrants in Thailand (4.4%) is up to 20 times that of Thai locals (0.2%);[4] and proximity to the Burma border is positively associated with malaria parasitemia [10,11]. Burma may represent a reservoir of infection for its neighbours, but few data exist on the magnitude of transmission among border populations of Burma.

There are two published estimates of malaria prevalence in eastern Burma. Overall *Pf *prevalence was 15.8% among a convenience sample of symptomatic Burmese villagers (*n *= 703) seeking care in Thailand in 2001 [11]. A cluster mortality survey conducted in a conflict zone of eastern Burma in 2004 estimated a 12.4% (216/1739) prevalence among asymptomatic villagers [13].

The goals of the present analysis are: 1) to describe the prevalence of *Pf *in an area of active conflict in eastern Burma; 2) to explore the epidemiology of *Pf *parasitemia by age, sex, and season; and 3) to compare prevalence estimates from observational malaria program data and retrospective mortality cluster surveys.

## Methods

### Population

In late 2004 there were an estimated 526,000 internally displaced persons (IDPs) in eastern Burma, and at least 240 villages had been destroyed, forcibly displaced or abandoned in the prior two years [14]. Conservative estimates of ongoing displacement suggest that an additional 167,000 people and 300 villages were forced to move in the two years subsequent to the 2004 report [15].

**Figure 1:**
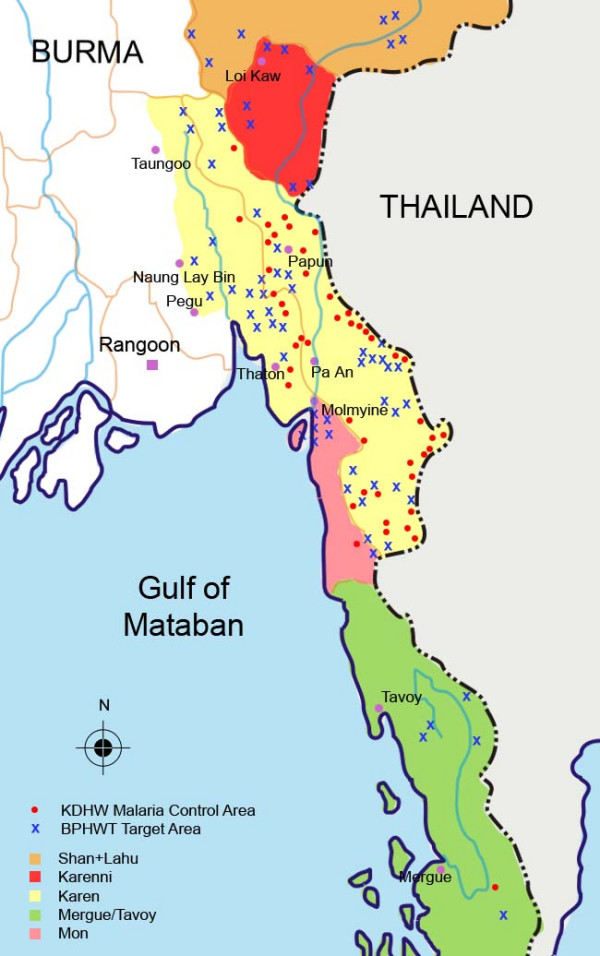
Target area of the KDHW and BPHWT. BPHWT: Backpack   Health Worker Team; KDHW: Karen Department of Health & Welfare.

Data in this study were collected from the so-called "black zones" in eastern Burma where health services are unavailable from either the military regime or international organizations. Services for a population of approximately 250,000 are provided primarily by ethnic health organizations of the Karen Department of Health and Welfare (KDHW) and the Backpack Health Worker Team (BPHWT), whose broad geographic target area extends from Mergui-Tavoy in the South to Karenni (Kayah) area in the North, and from the Thai-Burma border to slightly west of the Sittang River in eastern Pegu (Bago) Division. (Figure 1) For the purposes of service delivery and health information the two populations are mutually exclusive, in that BPHWT was designed to serve populations unable to access ethnic health clinics due to distance and/or security.

KDHW administers 33 clinics to provide primary health care to approximately 95,000 persons. These semi-permanent clinics are located in relatively stable areas of Karen State, but are designed for rapid relocation in the case of threats to population security. Eleven clinics have been forced to relocate since 1998, five from October 2006 to April 2007. BPHWT is comprised of over 300 health workers divided into 76 teams designed to reach an additional 152,000 persons in less stable areas. Since inception of the program in 1998, seven BPHWT health workers have died while carrying out their health care provision responsibilities.

This report summarizes and compares *Pf *prevalence estimates derived from two types of data sources: cross-sectional screenings conducted as part of the KDHW malaria program from 2003 to 2006, and retrospective cluster surveys designed to estimate infant mortality rates in the entire BPWHT and KDHW populations in 2004 and 2006. Both the malaria program and the cluster surveys identified *Pf *parasitemia with a rapid diagnostic device (RDT; Paracheck-Pf^® ^Orchid Biomedical Systems, Goa, India).

### Integrated Malaria Control Program

In 2003 the KDHW initiated an integrated malaria control program in four villages with a total population of 1,819. By 2006 the program reached 28,498 persons in 49 villages (village population size range: 162 – 1,824). This population is a subset of the entire KDHW population of 95,000. The pilot program included distribution of long-lasting insecticide treated nets (LLITNs), malaria education messages, and early detection with the Paracheck-Pf^® ^device and therapy with mefloquine-artesunate for three days (MAS3). Baseline screenings were conducted prior to initiation of malaria control activities, permitting the estimation of malaria prevalence among new villages in each year.

The decision to actively screen a population living in an area of unstable transmission was based on the dramatic success of a similar strategy in Vietnam [16,17] and later in Brazil [18,19] and Cambodia [20]; and on growing, albeit inconsistent, evidence for asymptomatic infections in areas of unstable transmission [21-23] including Burma [24,25].

Screening was universal in the first phase of the program (2003–2004). However, in order to reduce costs, limited screening was conducted in 10 of 14 new villages in 2005, and all new villages in 2006 (N = 27). Limited screenings included a systematic sample of 100 heads of household. Females were preferentially sampled during limited screenings in order to minimize the workload of health workers operating in a conflict zone, and to maximize the likelihood of identifying parasitemia in women of reproductive age. Villages with fewer than 100 households in 2006 (N = 11) screened only one person per household. All participants with a positive test result in either the malaria program or the cluster surveys (described below) received MAS3, as recommended by regional guidelines [26].

Parasitemia prevalence is reported as the proportion of the screened population with a positive Paracheck-Pf^® ^test result [(number *Pf *positive)/(total number screened)]. Estimates in 2005 and 2006 were weight-adjusted by village population size. Confidence intervals for prevalence estimates were calculated for finite populations to account for near-complete sampling by multiplying the standard error by the square root of (1 - *p*), where *p *is the proportion of the population that is sampled [CI = +/- 1.96 * SE(1−p)
 MathType@MTEF@5@5@+=feaafiart1ev1aaatCvAUfKttLearuWrP9MDH5MBPbIqV92AaeXatLxBI9gBaebbnrfifHhDYfgasaacH8akY=wiFfYdH8Gipec8Eeeu0xXdbba9frFj0=OqFfea0dXdd9vqai=hGuQ8kuc9pgc9s8qqaq=dirpe0xb9q8qiLsFr0=vr0=vr0dc8meaabaqaciaacaGaaeqabaqabeGadaaakeaadaGcaaqaaiabcIcaOiabigdaXiabgkHiTiabdchaWjabcMcaPaWcbeaaaaa@31BF@].

Prevalence estimates from the eleven villages conducting universal screening were stratified by sex and age (<5, 5–14, and 15+ years), and crude odds ratios and their 95% confidence intervals were calculated. The rainy season was defined as the 5 months from June through October to account for parasite development in the mosquito following the onset of the rainy season between May and early October.

### Cluster Survey Design

This report included results from four retrospective mortality cluster surveys conducted in two different years in the two mutually exclusive target populations of the BPHWT and KDHW. Between October and December in 2004 and 2006, BPHWT and KDHW health workers conducted retrospective household surveys of vital events and human rights violations occurring in the 12 months prior to the interview. The design, implementation, and operational method of the surveys have been described previously [13,27]. Briefly, in 2004 and 2006 annual village census information was used to construct a sampling frame for the target population (~130,000) and spanning eight administrative areas (Figure 1). In 2004, one hundred village-based clusters (200 in 2006) were selected proportionate to population size and twenty (10 in 2006) households within each cluster were selected using systematic interval sampling. Design and implementation of surveys in KDHW areas differed only in the size of the sampling frame (~95,000).

At each household, surveyors explained the objectives and obtained verbal consent for participation. The survey included a listing of all household members by age and sex, and documented *falciparum *malaria parasitemia for the respondent using the Paracheck-Pf^® ^device.

### Sample Size and Analysis of Cluster Surveys

The proposed sample size for each survey was based on a balance of operational feasibility and resource constraints and the goal of continued monitoring of the infant mortality rate. Population proportions were estimated for several morbidity outcomes, including the proportion of respondents testing positive for *Plasmodium falciparum*. All confidence intervals were adjusted for the cluster sampling. The sample size allows for the estimation of parasitemia prevalence to within 2%, assuming baseline prevalence = 10%, overall survey completion rate = 85%, and design effect = 2.0.

### Ethical Approval

Data were collected as part of routine program monitoring and evaluation. Data forms were brought from the field to Mae Sot, Thailand where they were entered into a computerized database (Microsoft ACCESS) and were cleaned using range and internal consistency checks. The survey protocol and malaria program data collection instruments were approved by local leaders of the Burma Medical Association. The Johns Hopkins University Committee on Human Research approved the secondary analysis of the cluster survey data. The authors of this paper were responsible for the secondary analysis, conducted with Stata 8.2 (Stata Corp., College Station, TX, USA).

## Results

### Malaria Program Screenings

Between 2003 and 2006 a total of 9,796 RDTs were performed among 28,410 villagers participating in 11 universal (*n *= 5,872) and 36 limited (*n *= 3,924) baseline screenings. Each baseline screening was completed in approximately 3 (median) days, (range 1–7). Overall participation in universal screenings was 98.1% (village range 87–100%) of the expected population. Overall 800 RDTs were positive for *Pf*, representing a weighted-mean prevalence of 9.5%, 95% CI: 8.7 – 10.2.

**Figure 2:**
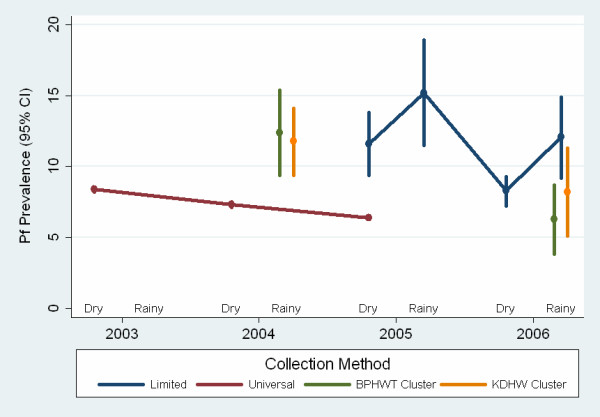
Estimates of *Plasmodium falciparum *prevalence from malaria program screenings and retrospective cluster surveys 2003 – 2006, by season. BPHWT: Backpack Health Worker Team; KDHW: Karen Department of Health & Welfare. Limited program screenings targeted female heads of household. Rainy season defined as the months from June – October.

Overall prevalence estimates derived from baseline universal and limited screenings in each year from 2003 to 2006 are presented in Figure 2. Prevalence in malaria program areas was similar over four years (2003: 8.4%, 95% CI: 8.3 – 8.6; 2004: 7.1%, 95% CI: 6.9 – 7.3; 2005:10.5%, 95% CI: 9.3 – 11.8 and 2006: 9.3%, 95% CI: 8.2 – 10.6).

There was substantial inter-village variation of *Pf *prevalence among villages (range 0% – 28.6%). In 2005, the only year that included both universal and limited screenings, combined prevalence in ten villages conducting limited screening (12.5%, 95% CI: 10.6 – 14.4%) was higher than in four universally screened villages (6.4%, 95% CI: 6.3 – 6.5).

### Age, Sex & Season

Universal screenings in 11 malaria program villages from 2003 to 2005 permitted comparison of *Pf *prevalence by age and sex (Table 1). Children under 5 years old (prevalence 9.6%) and children 5 to 14 years old (10.8%) had approximately twice the odds of testing positive (respective ORs: 1.99, 95% CI: 1.93 – 2.06; 2.24, 95% CI: 2.18 – 2.29) as adults 15 years or older (prevalence 5.1%). Prevalence was slightly higher among males (7.4%) than females (7.1%), though the difference overall was small (0.3%; OR 1.04, 95% CI: 1.02 – 1.06) and was attributable to a difference between male (5.5%) and female (4.7%) adults (OR 1.18, 95% CI: 1.14 – 1.21).

**Table 1:** *Plasmodium falciparum *prevalence from baseline universal screening in KDHW malaria control program villages (2003–2005), by age and sex

					**Crude OR (Finite Population 95% CI)**			
		**N**	***Pf *(+)**	***Pf *(%)**	Sex*		Age**	
Age <5								
	Male	499	48	9.6%	0.99	(0.93 – 1.06)	1.99	(1.93 – 2.06)
	Female	486	47	9.7%				
Age 5–14								
	Male	710	75	10.6%	0.97	(0.93 – 1.01)	2.24	(2.18 – 2.29)
	Female	721	79	11.0%				
Age 15+								
	Male	1,800	99	5.5%	1.18	(1.14 – 1.21)	(--)	
	Female	1,655	78	4.7%				
All Ages								
	Male	3,009	222	7.4%	1.04	(1.02 – 1.06)	(--)	
	Female	2,862	204	7.1%				

* Reference category is female within age category

**Reference category is individuals 15 years and above

Limited screening with 1,054 RDTs among 5,449 predominantly female (80 – 98%) heads of household in 10 villages in 2005, and 2,870 RDTs among 17,602 in 27 villages in 2006 facilitated the evaluation of the association of *Pf *prevalence with rainy and dry season. (Table 2) Prevalence was higher in the rainy season than the dry season in both 2005 (weighted prevalence 15.2% vs. 11.6%) and 2006 (12.4% vs. 8.3%; combined 2005–2006 OR 1.48, 95% CI: 1.16 – 1.88).

**Table 2:** *Plasmodium falciparum *prevalence estimated from limited screening* in KDHW malaria program villages (2005–2006), by season

	**2005**		**2006**					
**Season**	**Dry**	Rainy**	**Dry**	Rainy**				
# Villages	7	3	20	7				
Population	4,004	1,445	11,923	4,306	Crude OR (Finite Population 95% CI)			
Rapid Tests Performed	735	319	2,246	624				
Proportion Female	0.90	0.81	0.98	0.84	Rainy Season		Year (2006/2005)	

Prevalence	11.6%	15.2%	8.3%	12.4%				
95% CI***	(9.4 – 13.8)	(11.5 – 18.9)	(7.3 – 9.4)	(9.3 – 15.0)	1.48	(1.16 – 1.88)	0.72	(0.58 – 0.89)

*Limited program screenings targeted female heads of household

**Rainy Season = June – October

***Confidence intervals adjusted for sampling without replacement within a finite population

### Cluster Survey Results

To estimate *Pf *parasitemia prevalence in the entire target population among female heads of household, in 2004 and 2006 the mobile workers of the BPHWT conducted a total of 1,834 and 1,614 household surveys, representing 92% and 90% of the respective target sample populations. A slightly lower proportion (83%) was returned from KDHW areas in 2004. Characteristics of the survey samples are summarized in Table 3. A total of 5,538 rapid tests for parasitemia were conducted in four cluster surveys, representing 80% of respondents overall. Overall 10.2% (range 6.3% – 12.4%) were positive (Table 3). Prevalence point estimates were lower in both BPHWT and KDHW areas in 2006 than in 2004, though the difference reached statistical significance only for BPHWT surveys. The KDHW 2006 sampling frame included seven clusters in malaria control program areas (n = 180) where the prevalence (1.7%) was lower than in non-MCP clusters (n = 1,267, prevalence 9.1%).

**Table 3:** Cluster Survey Target Population, Response Rate and *Pf *Prevalence

	**2004**		**2006**	
**Sample Characteristic**	BPHWT*	**KDHW**	**BPHWT**	KDHW**
Target Population	129,000	96,888	134,732	89,092
Number of Clusters Sampled	100	100	180	100
Number of Clusters Successfully Reached	92	84	164	88
Total Households Sampled	1,834	1,657	1,614	1,835
Response Rate	92%	83%	90%	92%
Number of Malaria Rapid Tests Performed	1,739	1,588	882	1,329
Proportion of Respondents Tested	95%	96%	55%	72%
Proportion Female	62%	92%	84%	99%
*Plasmodium falciparum *Prevalence	12.4%	11.8%	6.3%	8.2%
Cluster Adjusted 95% Confidence Interval	(9.4 – 15.4)	(9.5 – 14.2)	(3.9 – 8.8)	(5.1 – 11.3)

BPHWT: Backpack Health Worker Team; KDHW: Karen Department of Health & Welfare

* BPHWT 2004 survey results reported previously in Mullany, Richards, Lee et al. (2007). See reference 13.

** KDHW 2006 sampling frame includes seven malaria control program areas (n = 180) with *Pf *prevalence of 1.7%. Prevalence in non-MCP clusters (n = 1267) was 9.1%.

## Discussion

The prevalence of *plasmodium falciparum *in conflict areas of eastern Burma prior to malaria interventions has remained high (at least 6.3% – 12.5%) over the four year period 2003–2006. Estimates are derived from over 15,000 rapid tests performed in a combined target population of over 225,000 persons and represent one of the largest samples reported from southeast Asia. The range of village prevalence (0 – 28.6%) is consistent with smaller reports from other areas of Burma (range 10–40%) [11,28-30]. The overall prevalence estimate presented here is higher than the prevalence of 3.9% (range 2–7%) documented in 2006 in four Burmese villages along the Thai border with ongoing malaria control efforts [31]. Prevalence in eastern Burma is also higher than that recorded among Thai villagers (prevalence <2%) and foreign nationals (<3.5%) in Thailand, [11] confirming the presence of a malaria reservoir in eastern Burma that likely contributes to transmission in border regions of Thailand.

### Age

The higher *Pf *prevalence we observed in children compared to adults is consistent with population surveys in ecologically similar areas of Laos, [32] Cambodia [20,23] and Burma. For example, Tun-lin et al. documented higher prevalence in children under ten (30%–50%) than in adults (10–27%) during four successive screenings (*n *= 146 – 168) in a single village in central Burma in 1992 – 1993 [28]. However, an HRP-II antigen assay might overestimate *Pf *prevalence in children relative to adults, as acquired immunity in adults might lead to lower parasitemia levels and may decrease the sensitivity of the antigen assay.

### Sex

We did not observe large differences in *Pf *prevalence between males and females in either children or adults. These results differ from the observation of a four-fold higher *Pf *prevalence among male (9%) vs. female (2%) adults in four Burmese villages immediately across the Thai border with access to early detection and treatment (EDT), [31] as well as from other studies southeast Asia that have documented increased exposure of male adults to infected mosquitoes due to forest related activities [23,28,33,34]. The discrepant observations may reflect a difference in forest-related behaviors or an influence of the location and/or stability of villages; but may also reflect the lack of access to EDT or other malaria control interventions prior to our surveys. The higher *Pf *prevalence in males noted in other studies may reflect the relative impact of malaria control programs in adult men and women, and may not reflect the sex distribution of asymptomatic *Pf *among adults prior to program implementation.

### Season

Overall prevalence was higher during the rainy season in both 2005 and 2006. This seasonal variability, however, appears to be less than that observed in *Pf incidence *among Burmese migrants [10] and refugees [35] in Thailand. These data are similar to those from bi-annual screenings conducted in four Burmese villages in 2006 (*Pf *prevalence 3.9% in both the rainy and dry seasons) in the setting of ongoing malaria control [31].

### Malaria Program Screenings vs. Cluster Surveys

In 2004, cluster surveys produced higher estimates of *Pf *prevalence (12.4% and 11.8%) than program areas (7.1%). There are several possible reasons for this discrepancy. In 2004, malaria program screenings included nearly the entire population, whereas cluster surveys screened only heads of households, who may be more likely to engage in behaviours with elevated malaria risk, such as forest-related activities. While we did not directly measure malaria risk behaviours, in universally screened malaria program areas we observed that adults were at significantly *lower *risk than children. Alternatively, the higher prevalence reported in the cluster surveys in 2004 may reflect differences in village location, stability and/or exposure to human rights violations. Studies have documented an increased risk of malaria among migrants [34,36] and in the setting of complex emergencies [7,37]. Results reported elsewhere [13] from the 2004 survey in BPHWT areas suggest that malaria prevalence may be associated at the household level with forced displacement, forced labour, and destruction of food supply, and that exposure to multiple human rights violations increases risk.

Location and stability may also have contributed to the higher prevalence observed in 2005 among malaria program villages conducting limited screening among heads of household (population weighted prevalence 12.5%, 95% CI: 7.5 – 17.5) compared to universally screened villages (6.4%, 95% CI: 6.3 – 6.5), which tended to be located among more stable populations. The villages with the highest prevalence in both 2004 (Mae Ngaw, 17%) and 2006 (Ei Tu Hta, 29%) were the least stable villages in those years. Mae Ngaw subsequently was destroyed by the military in early 2005 and Ei Tu Hta was a newly formed encampment for persons internally displaced in early 2006 by an escalation of violence near the new Burmese capital of Pyinmana (Naypyidaw).

### Limitations

Sequential estimates of *Pf *prevalence in new malaria program areas perforce relied on screening different villages in each term, which likely resulted in substantial bias by area and other unmeasured factors. An alternative approach to include longitudinal measurements in intervention-naïve villages would have minimized this bias, but was not possible in this setting, as implementing partners felt it would be unethical to withhold effective interventions from vulnerable populations. Furthermore, the increasingly large number of areas included in screenings, as well as triangulation with estimates from cluster sample surveys, enhances the external validity of our findings to other villages in "black zones" of eastern Burma.

We did not conduct universal screenings in all villages. However, the number of RDTs from universal screenings performed from 2003–2005 (*n *= 5,871) permitted evaluation of associations with age and sex; and limited screening resulted in significant cost savings to facilitate program expansion to additional villages. In universally screened villages, the *Pf *prevalence overall was higher (7.2%) than the estimate among adult women (4.4%) and this relationship was consistent for each year in which universal screening was conducted. This suggests that the population prevalences in cluster surveys and in program areas conducting limited screening, where adult women were over-sampled, likely underestimate the true population-based burden of parasitemia.

The use of a rapid diagnostic test may have limited our ability to detect low level parasitemia [38-40]. However, Paracheck-Pf^® ^has demonstrated impressive sensitivity and specificity under field conditions during asymptomatic screening of children in India (sensitivity/specificity 94.4 & 89.0%, respectively), [41] and in Tanzanian villages with either high (40.1%), low (4.3%), or very low (1.9%) *P. Falciparum *prevalence (sensitivity 83.6, 100%, n/a; specificity 94.1%, 99.5%, 98.4% respectively) [42,43]. Furthermore, the alternative diagnostic strategy in areas where PCR is unavailable – field microscopy – has shown poor sensitivity (~10%) for asymptomatic *P. Falciparum *parasitemia [22] when compared to expert microscopy in western Thailand, [44] suggesting Paracheck-Pf^® ^may be at least as accurate as field microscopy in this setting. The accuracy of RDTs may be compromised by high-temperatures or prolonged storage under field conditions, [42] but storage in thatched huts likely minimized extreme temperatures in our case. Low RDT sensitivity would have resulted in an underestimation of parasitemia prevalence. It is unlikely that false positives (due to low specificity) accounted for a high proportion of prevalent cases during baseline screenings, given the consistently low prevalence (<2%) recorded during follow-up in most malaria program areas during program implementation [45].

Although most participants were asymptomatic at the time of testing, those with a positive RDT were treated immediately; therefore we are unable to distinguish between pre-clinical and chronic asymptomatic infection. Other studies from Burma, [24,25] Cambodia, [23] Tanzania [43] and South America [18,21,46] suggest that protective immunity (premunition) is not uncommon in the setting of unstable transmission; and that asymptomatic infection is infectious to mosquitoes despite a low asexual parasite burden [46-48]. The relatively high prevalence of predominantly asymptomatic parasitemia in this report adds to the growing body of evidence supporting the presence of asymptomatic infection in areas of unstable malaria transmission. Additional studies are needed to estimate the prevalence of asymptomatic carriers in eastern Burma and to evaluate the role of active case detection in reducing malaria transmission.

We did not estimate the prevalence of *plasmodium vivax *(*Pv*), though *Pv *appears to account for no more than 20% of malaria infections in Burma [6] and almost certainly represents an even smaller fraction of malaria related deaths. We did not directly measure rainfall, [49] migration, [36] forest-related activity, proximity to water [50] or other risk factors for malaria [51] that may have confounded the associations we observed.

Rapid testing with accurate RDTs is easily integrated into malaria control programs and cluster surveys designed to estimate other health indicators, and provides a simple and cost-effective means to estimate cross-sectional prevalence of parasitemia. Triangulation of data from different sources enhances the validity of parameter estimates. Additional studies are necessary to quantify malaria risk in eastern Burma, including the role of age and sex, elevation, season, migration, forest-related activities and civil conflict. Increasing capacity for EDT offers an opportunity to directly monitor the more clinically relevant incidence of symptomatic *Pf*, and to improve our understanding of the relationship between *Pf *incidence and prevalence in this setting. In a region with highly drug resistant *Pf *[2,52] and ubiquitous fake antimalarials [3,53] efforts to track treatment failures and to monitor *in vitro *drug susceptibility and antimalarial quality should be expanded in unstable areas in eastern Burma.

## Conclusion

Prevalence of *plasmodium falciparum *in a large population in conflict areas of eastern Burma remains high relative to the prevalence reported among populations in neighboring Thailand, particularly among children. There is an immediate need to expand malaria interventions to reduce morbidity and mortality in conflict areas in eastern Burma and to reduce the reservoir of infection that compromises regional disease control efforts.

## Abbreviations

**KDHW: **Karen Department of Health and Welfare

**BPHWT: **Backpack Health Worker Team

***Pf: ****plasmodium falciparum*

***Pv: ****plasmodium vivax*

**IDPs: **internally displaced persons

**LLITNS: **long-lasting insecticide treated nets

**MAS3: **mefloquine-artesunate combination therapy for 3 days

**RDTs: **rapid diagnostic tests

**EDT: **early diagnosis and treatment

**MCP: **Malaria Control Program

**CI: **confidence interval

## Competing interests

The author(s) declare that they have no competing interests.

## Authors' contributions

AR conceived the study, participated in the design of the malaria control program and cluster surveys and in the management of study data, was responsible for the statistical analysis and interpretation of the data and drafted the manuscript. LS and LM participated in design of the malaria program and cluster surveys, took primary responsibility for data management, and assisted in analysis and interpretation of the data and revision of the manuscript. CL participated in design of the malaria program and cluster surveys and assisted in management, analysis and interpretation of the data. EW participated in the design of the malaria control program and cluster surveys and the revision of the manuscript. KB participated in the design of the malaria control program, assisted with interpretation of the data, and participated in revision of the manuscript. MM directed the implementation of the cluster surveys and was responsible for quality control in BPHWT areas, and assisted with interpretation of the data. ES designed and implemented the malaria control program, conducted the cluster surveys and was responsible for quality control in KDHW areas, and assisted with interpretation of the data. TL participated in the design of the malaria control program and cluster surveys, in the management and interpretation of study data and in revision of the manuscript. All authors read and approved the final manuscript.
